# Induced and natural variation affect traits independently in hybrid *Populus*

**DOI:** 10.1093/g3journal/jkae218

**Published:** 2024-09-12

**Authors:** Weier Guo, Héloïse Bastiaanse, Julin N Maloof, Luca Comai, Isabelle M Henry

**Affiliations:** Genome Center and Department of Plant Biology, University of California Davis, Davis, CA 95616, USA; Genome Center and Department of Plant Biology, University of California Davis, Davis, CA 95616, USA; Department of Plant Biology, University of California Davis, Davis, CA 95616, USA; Genome Center and Department of Plant Biology, University of California Davis, Davis, CA 95616, USA; Genome Center and Department of Plant Biology, University of California Davis, Davis, CA 95616, USA

**Keywords:** dosage variation, QTL, poplar, natural variation, trait, Plant Genetics and Genomics

## Abstract

The genetic control of many plant traits can be highly complex. Both allelic variation (sequence change) and dosage variation (copy number change) contribute to a plant's phenotype. While numerous studies have investigated the effect of allelic or dosage variation, very few have documented both within the same system, leaving their relative contribution to phenotypic effects unclear. The *Populus* genome is highly polymorphic, and poplars are fairly tolerant of gene dosage variation. Here, using a previously established *Populus* hybrid F1 population, we assessed and compared the effect of natural allelic variation and induced dosage variation on biomass, phenology, and leaf morphology traits. We identified QTLs for many of these traits, but our results indicate limited overlap between the QTLs associated with natural allelic variation and induced dosage variation. Additionally, the integration of data from both allelic and dosage variation identifies a larger set of QTLs that together explain a larger percentage of the phenotypic variance. Finally, our results suggest that the effect of the large indels might mask that of allelic QTLs. Our study helps clarify the relationship between allelic and dosage variation and their effects on quantitative traits.

## Teaser

Both environmental and genetic variation can affect plant growth. Within the genetic factors, both DNA sequence and dosage (gene copy number) can change and affect plant function. Here, Guo *et al*. use a population of poplar trees to investigate if these two types of changes affect plants similarly, and whether or not they interact with each other. This helps create a broader understanding of what matters most to proper plant function and what is most likely to affect it.

## Introduction

Natural allelic variation plays an important role in phenotypic diversity in plants (Alonso-Blanco *et al.* 1999, 2009; Todesco *et al.* 2010; Huang *et al.* 2011, 2012; Huang and Han 2014; Jin *et al.* 2016; Satbhai *et al.* 2017; Zhang *et al.* 2021; Duan *et al.* 2022). The statistical framework raised by R. A. Fisher provides an approach to systematically identify the quantitative trait loci (QTL) responsible for heritable variation (Fisher 1919). In the last decade, the development of new DNA high-throughput sequencing and genotyping technologies has dramatically improved our ability to identify polymorphic genetic markers between individuals or species (Gupta *et al.* 2008; Davey *et al.* 2011; Elshire *et al.* 2011). This, in turn, enables more accurate QTL identification in both plants and animals (McMullen 2003; Wellcome Trust Case Control Consortium 2007; Rafalski 2010; Jamann *et al.* 2015). Despite these technological advances, a wide percentage of the observed phenotypic variance still remains unexplained by the detected QTLs. This is particularly problematic for complex traits with expected polygenic contributions. For example, the QTLs detected through the analysis of biomass-related traits in *Populus* explain, on average, 26% of the observed phenotypic variation (Rae *et al.* 2009). To increase biomass yield through tree breeding, we need to consider other types of heritable variations, aiming for a deeper understanding of the underlying regulatory mechanisms.

Besides allelic variation (sequence variation that does not involve copy number changes), dosage variation can also affect the phenotypic outcomes of many important plant traits. Copy number variation (CNVs), especially the ones affecting protein-coding regions, have been associated with phenotypic outcomes in multiple plant species (Cook *et al.* 2012; Díaz *et al.* 2012; Li *et al.* 2012; Carbonell-Bejerano *et al.* 2017; Prunier *et al.* 2019). Pan-genomic analyses have identified structural variants across different accessions of multiple plant species, many of which affect important agronomic traits such as flower size, fruit weight, and heat tolerance (Golicz *et al.* 2016; Pinosio *et al.* 2016; Alonge *et al.* 2020; Zmienko *et al.* 2020; Yan *et al.* 2023). Gene deletion and duplication can directly affect expression level (*cis*-effect), which in turn affects phenotypes. Gene dosage may also affect phenotype through mechanisms explained by the gene balance hypothesis (Birchler and Veitia 2012). Dosage variation can also modulate the expression of genes located outside of indel regions (*trans*-effect), since many traits are regulated by a complex network comprising multiple genetic components (Birchler and Veitia 2010; Veitia *et al.* 2013).

To increase our understanding of the relative contributions of these two sources of phenotypic variation, we investigated the phenotypic effects of induced dosage variation and natural allelic variation within the same population. We also aimed to document instances of interplay between these two sources of variation. For example, when a locus encodes a protein whose function is dosage sensitive, the CNV-induced expression changes affect the phenotype. However, if allelic variation is also present, such as if one allele is hypomorphic or null, two scenarios are possible: (1) the CNV affecting the deficient allele results in no or little phenotypic variation or (2) the CNV affecting the normal allele results in magnified phenotypic variation. Either way, focusing on either the allelic variation or the dosage variation alone only addresses part of the mechanisms at play. A more comprehensive approach, which integrates both types of variations may be better suited to fully understand the genetic regulatory factors of complex traits.

*Populus* is an attractive system to study the interplay between allelic and dosage variation. It is dioecious and therefore an obligate outcrosser and its genome are highly polymorphic, both in terms of sequence polymorphisms and CNVs (Tuskan *et al.* 2006; Pinosio *et al.* 2016). Pollen irradiation is a widely used approach for inducing indel mutations in plants (Brewbaker and Emery 1961; Yang *et al.* 2004), starting as early as the 1950s (Nuffer 1957; Mottinger 1970). In tree species, pollen irradiation followed by pollination has been well-established (Osborne 1957; Rudolph 1978). Gamma-induced indels, especially larger ones, are not typically retained in future generations because they are often associated with lethality in the gamete, where the copy number goes down to zero. In clonally propagated crops such as *Populus*, on the other hand, they can be retained indefinitely. In a previous report, we described the establishment of a *Populus* F1 hybrid population (592 lines) from an interspecific cross between a wild-type *P. deltoides* mother and gamma-irradiated pollen from *P. nigra* (Henry *et al.* 2015b). Whole-genome sequencing analysis revealed that 58% of the F1 lines carry large-scale insertions or deletions (indels). The size of induced indels varies from 250 kb to whole chromosomes. The number of indels per line varies between 0 and 10, with 2.5 indels per individual on average. Indels from different lines can overlap such that each genomic region is covered by 1–31 indels and only 1.6% of the genome (6.2 Mb) is not covered by any indel at all.

Using this resource, we investigated the association between dosage variation across the genome and a variety of phenotypes. This resulted in the identification of “dosage QTLs” associated with biomass, phenology, leaf morphology, and vessel development traits (Bastiaanse *et al.* 2019, 2020a; Rodriguez-Zaccaro *et al.* 2021). Since both parental genomes are highly polymorphic, natural allelic variation is expected to play an important role in the observed phenotypic variation, but it was not taken into account in these earlier studies.

**Fig. 1.**
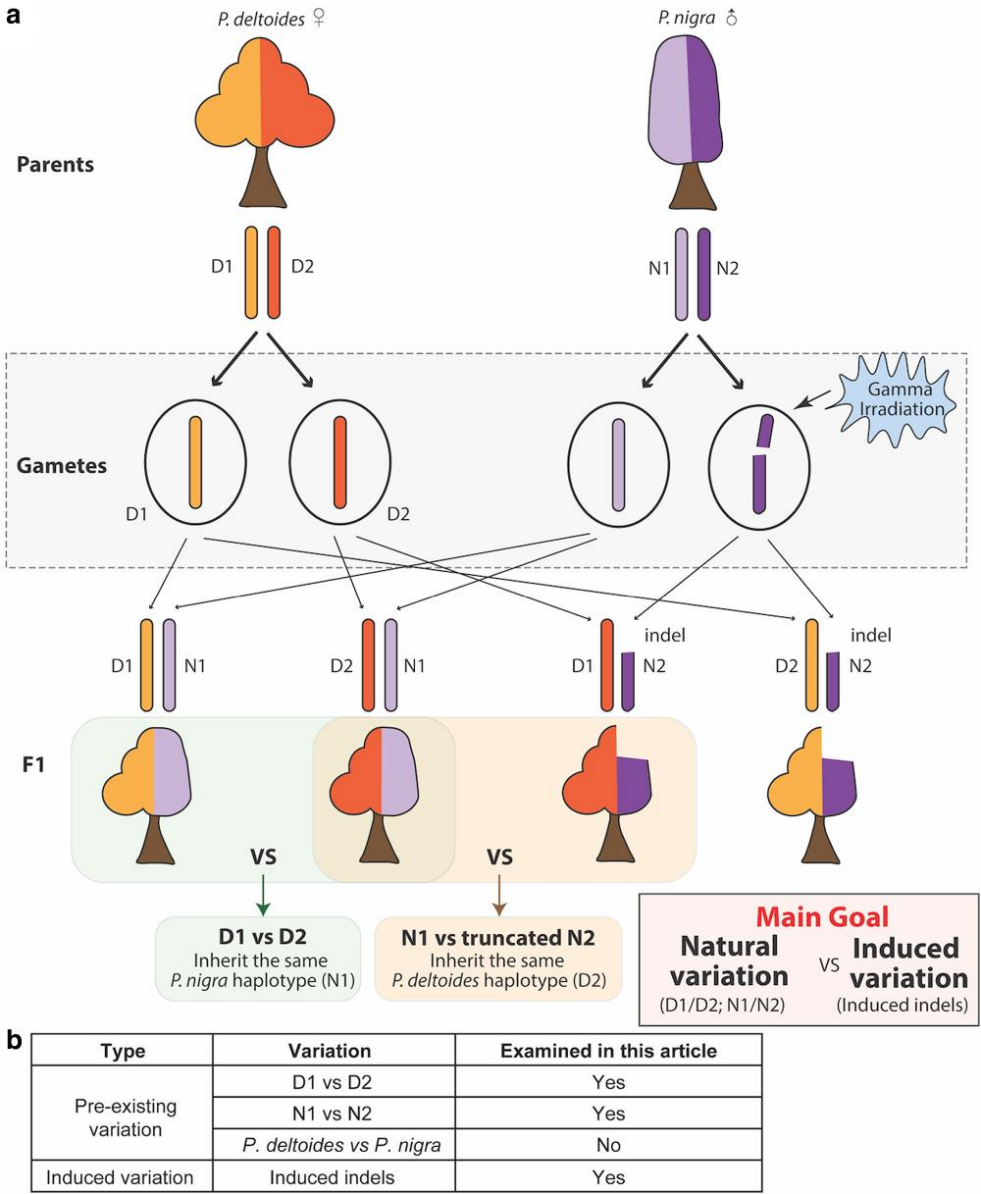
Major goal of this study. a) Illustration of the *Populus* population used in this study and the main goal of this study. The F1 population came from an interspecific cross between *P. deltoides* (female) and *P. nigra* (male). The phenotypic differences can result from i) natural variation (D1/D2, N1/N2); ii) Radiation-induced variation (indels). Our main goal is to investigate the interplay between natural variation and induced variation. D1/D2: *P. deltoides* haplotype1 and haplotype2. N1/N2: *P. nigra* haplotype1 and haplotype2. b) Type of variation examined in this article. For natural variation, we are examining the SNPs between 2 haplotypes within *P. deltoides*, as well as the SNPs between 2 haplotypes within *P. nigra*. We are not testing the species-specific SNPs (*P. deltoides* vs *P. nigra*) in this study. For induced variation, we examined the radiation-induced indels.

Here, we aim to investigate whether allelic variation, and in this case, the differences between the two haplotypes within each parent, also influence these traits (allelic QTLs). Next, we aimed to document the possible interaction between natural allelic variation and induced dosage variation in this population (Fig. 1). This *Populus* clonal system is superior to our study goal since it allows us to obtain replicated phenotypic information easily. In a subset of 343 F1 lines, all offspring of the same two parental clones from this *Populus* population, and detected both dosage and allelic QTLs. Our results suggest a limited overlap between QTLs associated with allelic and dosage variation. A custom method was developed to assess the effect of both allelic and dosage variation in a joint model. The results indicated that allelic and dosage variation affect traits independently. Detection of allelic QTLs in a subset of the population that does not carry large indels resulted in a different set of QTLs, suggesting that large-scale indels might mask the effect of allelic QTLs in the full population. Finally, direct integration of both types of QTLs makes the association between trait values and genetic information stronger.

## Materials and methods

### Data acquisition and preprocessing

Genomic sequencing data, RNA-seq data, and phenotypic information were obtained from previous studies (Henry *et al.* 2015b; Zinkgraf *et al.* 2016; Bastiaanse *et al.* 2019, 2020a). Briefly, an interspecific cross between wild-type *P. deltoides* and pollen-irradiated *P. nigra* produced 592 F1 hybrid lines. High-coverage Illumina short-read sequences were obtained from the two parental lines with read depth around 45× and 65× for *P. deltoides* and *P. nigra*, respectively. Additionally, low-coverage Illumina genome sequences were obtained from each of the F1 hybrid clones (read depth around 0.5× per line). Leaf RNA sequencing was performed on 166 F1 lines, each in triplicates. The raw RNA-seq reads were pooled per clone and used to assist in haplotype phasing. The collection and statistical analysis of phenotypic information were described in previous studies (Bastiaanse *et al.* 2019, 2020a). Three categories of phenotypes—leaf morphology, phenology, and biomass—were used in our study (Supplementary File 1).

The preprocessing of sequencing data followed a custom pipeline developed previously. It starts with a demultiplexing step performed using a custom pipeline (https://github.com/Comai-Lab/allprep) for separating raw reads into individual libraries. Reads were aligned to the *Populus* reference *P. trichocarpa* v3.0 (Tuskan *et al.* 2006), using a custom Python script based on Burrows-Wheeler Aligner (Li and Durbin 2009) (https://comailab.org/data-and-method/bwa-doall-a-package-for-batch-library-processing-and-alignment/). Bam files were generated in this step, which were used to obtain a mpileup file using a custom Python package (https://github.com/Comai-Lab/mpileup-tools) based on Samtools (Li *et al.* 2009), followed by a simplification step to convert the mpileup file into a parsed-mpileup file.

### Haplotype phasing

To describe the parental haplotypes, we identified heterozygous positions in each parent and determined the phasing between these positions, using a custom computational pipeline (https://github.com/guoweier/QTL_manuscript). Specifically, we started by identifying single nucleotide polymorphisms (SNPs) that can distinguish between two haplotypes within a parent (Supplementary Fig. 1a). In short, we selected two lists of SNPs, one for *P. deltoides* and the other for *P. nigra*. The example of *P. deltoides* SNPs selection is shown in Supplementary Fig. 1a. For *P. deltoides*, we selected positions that exhibited heterozygosity in *P. deltoides* and homozygosity in *P. nigra*; or positions that showed heterozygosity in *P. deltoides* with different heterozygous allele combinations in *P. nigra*.

Next, we used RNA-seq data obtained from a subset of 122 F1 individuals to derive phased parental haplotypes (Supplementary Fig. 1b). Briefly, we first used the RNA-seq raw data from the diploid F1 lines for haplotype phasing, after retaining the positions that are at least 20× read depth in the RNA-Seq data. Second, we treated RNA-seq raw data as genomic sequencing data, with the preprocessing approaches that have been described above. Parsed-mpileup file with 122 RNA-seq lines was obtained after running the pipeline. Then, the RNA-seq parsed-mpileup file was used to identify inherited alleles from *P. deltoides* and *P. nigra*, respectively. Finally, we collected the adjacent SNPs combination orders and recorded the order as parental haplotypes when data from more than 90% (109 out of 122) of RNA-seq lines were consistent with it.

### Genotyping

The adjusted phased haplotypes were applied to low-coverage sequencing data for genotyping. Specifically, for each SNP marker, genotype in F1 hybrids was only recorded when it inherited the alternative allele. Recorded genotypic information was then binned (50 SNPs per bin) to increase the robustness of genotype calls. As a control, the same genotyping process was applied to the RNA-seq data. The transcriptomic genotypes and genomic genotypes were compared manually (all resulting figures can be viewed at https://github.com/guoweier/QTL_manuscript). Next, for the individuals for which both genomic and RNA-seq data were available, we sorted the F1 lines based on the read-depth of the low-coverage genome sequencing data. We then selected a read-depth threshold based on the following: a) Genotypes based on the low-pass genomic data clearly show an expected pattern of recombination along the whole genome and, b) Genotypes obtained from the genomic and RNA-Seq data are consistent. Lines for which only genomic data was available were retained if genomic coverage was above this threshold. As a result, 343 lines were selected to proceed for QTL analysis. Transcriptomic and genomic genotypes comparison of chromosome 1 on the selected F1 line with the lowest read-depth is shown in Supplementary Fig. 2.

### Dosage variation quantification

**Fig. 2.**
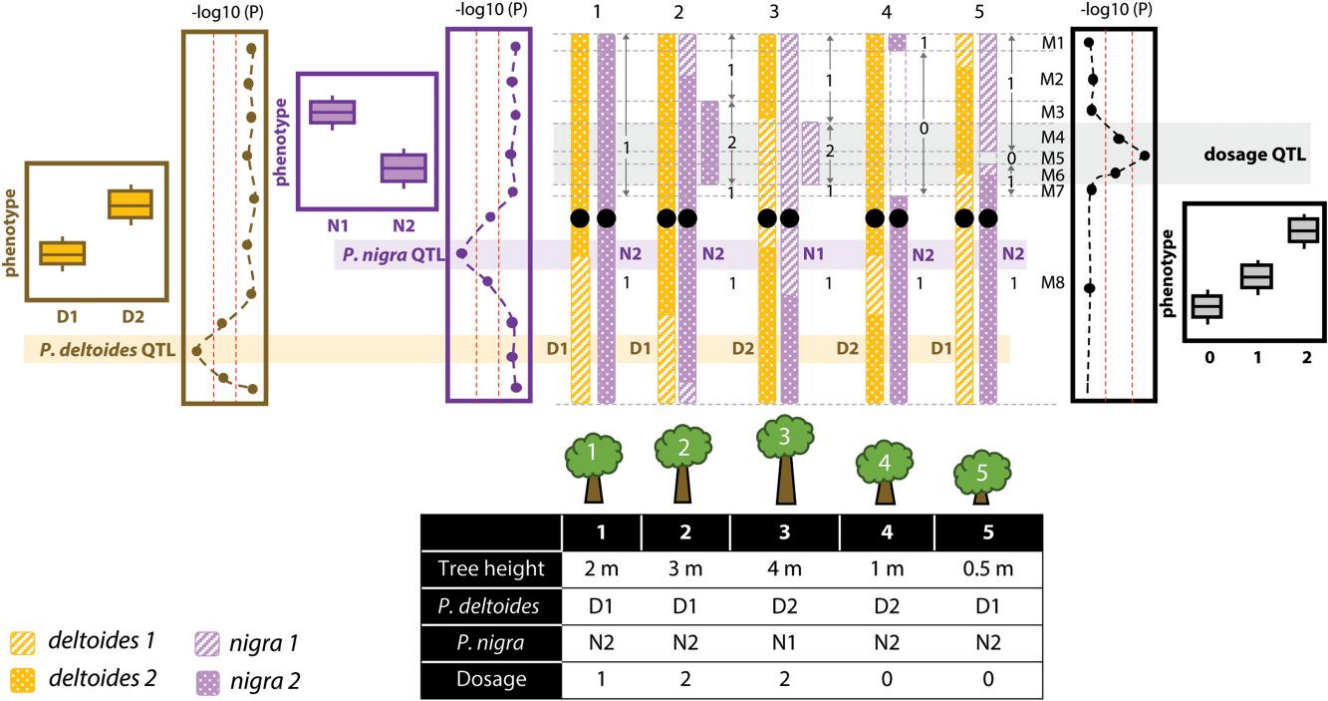
Representative illustration of QTL analysis using both allelic and dosage variation information. QTLs detected from sequence variation between two haplotypes (D1/D2) of *P. deltoides*, sequence variation between two haplotypes (N1/N2) of *P. nigra* and dosage variation can all contribute to the same trait (here tree height). *P. deltoides* and *P. nigra* haplotypes were acquired through analysis of allelic variation within each parent (D1/D2 or N1/N2). Dosage information was obtained through the calculation of relative copy number states in each chromosome bin (see details in Material and Methods).

Methods for quantifying dosage variation have been described in previous studies (Bastiaanse *et al.* 2019). Shortly, we defined bins based on indels breakpoints and tiled bins along the chromosomes. For each bin, the dosage genotype was determined by comparing the mean read coverage for each individual to the mean of the population. Dosage indicates the total copy number in any given bin. These F1 lines are diploids, so the background dosage number is 2. Since all dosage variation originates from *P. nigra* (Henry *et al.* 2015b), which is the paternal parent, we decided to only focus on the dosage changes in *P. nigra*. So the normal dosage state is 1, representing the F1 line carrying 1 copy from *P. nigra*. If an F1 clone carries a deletion which occupies 4 bins on chromosome 10, the dosage genotype for these 4 bins was set to 0, while the rest of bins on chromosome 10 were set to 1. Dosage genotypes were acquired for all 343 lines for which SNPs genotypes were also obtained. An illustration diagram can be found in Fig. 2.

### QTL analysis

To conduct a QTL analysis that simultaneously includes both allelic and dosage variation we employed a custom Python pipeline available at https://github.com/guoweier/QTL_manuscript. We generated a common marker list encompassing three types of variation: the *P. deltoides* haplotype, the *P. nigra* haplotype, and the dosage variation. First, we identified the physical positions of binned markers in *P. deltoides* and *P. nigra* genotypes, respectively. We then imputed genotypes in the unknown regions using information from their flanking binned markers. For example, on the *P. nigra* genotype, marker 1 is Chr01_1_10000 with genotype N1 and marker 2 is Chr01_20000_30000 with genotype N1. So the genotype in Chr01_10001_19999 is N1. If two flanking markers contained different genotypes, or if there was a missing flanking marker, the genomic region in between was assigned as a missing value “NA”. Second, we built a common marker list for the two parents, using *P. deltoides* markers as the reference and imputed *P. nigra* genotypes based on the markers' physical positions. Last, we applied the common marker to the dosage genotype and obtained the dosage value for each new marker.

Single models were established for analyzing the correlation between phenotypes and each variation type. The model is specified as follows:


$$Y_{i}=\beta_{0}+\beta_{1}gt_{i}+\varepsilon_{i}$$

where *Y_i_* is the phenotype; *β_0_* is the intercept; *β_1_* is the unknown coefficient; *gt_i_* is one of the examining genotypes (*P. deltoides* haplotype or *P. nigra* haplotype or dosage); and *ε_i_* is the residual variance. *P. deltoides* haplotypes were recorded as D1 or D2. *P. nigra* haplotypes were recorded as N1 or N2, while deleted regions were recorded as “NA”. The dosage of the *P. nigra* allele was recorded as 0 (deletion), 1 (regular), or 2 (insertion). To establish a suitable threshold for identifying significant QTLs, we employed a permutation test approach (Doerge and Churchill 1996). In short, for each trait and each genotype (*P. deltoides* haplotype or *P. nigra* haplotype or dosage), the phenotype data from the 343 F1 lines were randomized. Next, a linear regression between trait values and marker values was calculated with all the markers along the genome. The maximum *t*-value was selected. This randomization process was repeated 1,000 times. Then, we selected the top 5 and 1% of maximum *t*-values. In the observed dataset, the markers with *t*-values larger than the 5% threshold were considered significant, and those larger than the 1% threshold were considered as confirmed. Adjacent significant markers were considered as belonging to the same QTL.

To investigate how much phenotypic variance can be explained by each single QTL, we performed the QTL mapping using a multivariate model including all markers located underneath that QTL and extracted the adjusted *R*-square values. For phenotypic variance explained by all QTLs associated with one trait, we took the most significant marker (marker with the largest *t*-value) underlying each QTL and ran a multivariate model including these selected markers. Integration of QTLs from allelic and dosage variation followed a similar approach. For each trait, we collected the most significant marker from each QTL and fitted these markers into a multivariate model. Adjusted R-square values were recorded.

We designed a custom approach to perform QTL mapping combining all three types of variation. In short, we collected the genotypic information (*P. deltoides* haplotype or *P. nigra* haplotype or dosage) and assigned a State for each combined genotype. There were 10 possible States for the combined variable (Supplementary File 2). Then, a linear regression was performed using the lm() function in R, which is specified as follows:


$$Y_{i}=\beta_{0}+\beta_{1}State_{i}+\varepsilon_{i}$$

where *Y_i_* is the phenotype of the *i*th individual; *β_0_* is the intercept; *β_1_* is the unknown coefficient; *State_i_* is the variable after combining the three genotypes (*P. deltoides* haplotype or *P. nigra* haplotype or dosage) information of the *i*th individual; and *ε_i_* is the residual variance. Next, we performed pairwise comparisons of all present States using the function pairwisePermutationTest() in the R package “rcompanion” (Mangiafico 2020). Each comparison pair was treated independently, which generated 45 comparisons (Supplementary File 2). For each comparison, the *P*-values were collected and adjusted using the Benjamini and Hochberg (BH) method (Benjamini and Hochberg 1995). Adjacent markers were considered to belong to the same QTL. Last we identified the pairs of States that were significantly different to infer the possible genetic factors underlying the observed phenotypic variation. Specifically, QTLs were classified into 6 groups: deletion, deletion + insertion, insertion, *P. deltoides*, *P. deltoides + P. nigra,* and *P. nigra* (Supplementary File 2). The proportion of phenotypic variance explained by this custom QTL approach was determined using a method similar to that described above for QTLs from single models.

### Differentially expressed gene analysis and GO enrichment analysis

Differentially expressed genes were identified and the ones located within allelic QTLs were recorded. For each QTL, extreme phenotypic mutants (10 and 90% quantile) were selected, excluding the indel mutants having an indel under the QTL bins. Differential expression analysis were performed using the limma-voom method (https://ucdavis-bioinformatics-training.github.io/2022-April-GGI-DE-in-R/data_analysis/DE_Analysis_with_quizzes_fixed). Specifically, the estimated read counts were filtered such that only genes having more than 10 reads per million in at least 80% of the libraries were retained. *P*-values were adjusted using the Benjamin–Hochberg method (Benjamini and Hochberg 1995). Genes located under the QTL bins and with adjusted *P*-value < 0.05 were retained. The annotation information from Phytozome (https://phytozome-next.jgi.doe.gov/info/Ptrichocarpa_v3_1) was added for each gene.

GO terms for *Populus* genes were obtained from Phytozome (https://phytozome-next.jgi.doe.gov/info/Ptrichocarpa_v3_1). Enrichment analysis was performed by comparing GO terms of genes present in QTL bins against the genes expressed in leaf tissue. GO terms were considered suggestively enriched if the adjusted *P*-value (BH method) < 0.1.

## Results

### Deriving combined genotype and dosage information from low-coverage genome data

The *Populus* F1 lines (592) were originally sequenced at a low read depth (∼0.5× per line), which was sufficient to identify large-scale indels but was not sufficient to reliably haplotype and genotype each individual (Howie *et al.* 2009; Williams *et al.* 2012; Martin *et al*. 2016; Hager *et al.* 2020). Fortunately, RNA-seq data from 122 of these F1 lines was also available, as well as Illumina short-read sequencing data from two parental lines (*P. deltoides* 45×, *P. nigra* 65×) (Henry *et al.* 2015b; Bastiaanse *et al.* 2020a). Using these resources, we designed a custom computational process to derive parental haplotypes and genotype the F1 lines for both parental contributions (Fig. 2 and Supplementary File 3; see Materials and Methods).

The process is divided into 3 steps: parental SNP detection, parental haplotype phasing, and genotyping. Because our population is an F1 population, polymorphisms between the two parental genomes are not informative. Instead, we characterized the 2 pairs of parental haplotypes separately. We first selected 37,556 and 33,035 positions that were heterozygous in the parental clones of *P. deltoides* and *P. nigra*, respectively. Next, we used the RNA-seq reads from 122 diploid F1 lines to derive phased haplotypes for a subset of these SNPs for the two parents separately. Finally, the phased haplotypes were applied to the low-coverage genomic data (∼0.5× per line) to genotype the remaining F1 individuals. In total, we were able to obtain reliable genotype information for 343 F1 lines (Supplementary Fig. 1c). Last, we generated binned markers (50 SNPs per bin) to increase genotype robustness, and a final common marker set of 507 binned markers was generated for multi-genotype QTL analysis that applied to both the *P. deltoides* and the *P. nigra* genomes (Supplementary Fig. 3).

In terms of dosage variation, among the 343 remaining F1 lines, 54.2% (186 out of 343) were previously characterized to carry at least one indel. Deletions were more prevalent (66.5%) than insertions (33.5%) among these indels, as observed in the original population (Henry *et al.* 2015b). As described previously, we characterized dosage variation in 546 dosage binned markers, with an average of 6 indels in each dosage marker (Bastiaanse *et al.* 2019, 2020a; Rodriguez-Zaccaro *et al.* 2021). Finally, these dosage markers were combined with the natural allelic information to obtain a unified marker list of 507 binned markers, for which we had gathered information about the *P. deltoides* haplotypes, the *P. nigra* haplotypes, and the dosage information for each of the 343 F1 individuals.

### Contributions of natural allelic variation and induced dosage variation on phenotypes can be assigned to QTLs

This population was previously characterized phenotypically (Bastiaanse *et al.* 2019, 2020a; Rodriguez-Zaccaro *et al.* 2021) for 3 phenotype categories (38 traits): leaf morphology (22 traits), phenology (7 traits), and biomass (9 traits; Supplementary File 1). In our subset of 343 F1s, using a single model (Trait ∼ Genotype), QTLs were observed for 27 traits. Specifically, 9, 6, and 86 QTLs were identified from *P. deltoides*, *P. nigra*, and dosage genotypes, respectively (Table 1 and Supplementary File 4). Of the dosage QTLs detected here, 77.9% (67 out of 86) were detected in the previous analysis as well (Supplementary Fig. 4; Bastiaanse *et al.* 2019, 2020a).

**Table 1.** QTLs obtained using the three single models.^l^

Category*^a^*	Traits*^b^*	Trait ∼ *P. deltoides^c^*			Trait ∼ *P. nigra^d^*			Trait ∼ Dosage*^e^*		
		# of QTL*^f^*	% explained by single QTL (µ ± σ)*^g^*	% explained by all QTLs*^h^*	# of QTL*^f^*	% explained by single QTL (µ ± σ)*^g^*	% explained by all QTLs*^h^*	# of QTL*^f^*	% explained by single QTL (µ ± σ)*^g^*	% explained by all QTLs*^h^*
Biomass	Coppicing_y1*^i^*	1	4.6 ± 0	4.6	0	NA	NA	2	6.1 ± 1.6	7.0
	Diameter_base*^j^*	0	NA	NA	0	NA	NA	1	3.4 ± 0	3.4
	Time_serie_diameter_breast_height*^k^*	0	NA	NA	0	NA	NA	4	3.7 ± 0.1	7.6
	Volume*^j^*	0	NA	NA	0	NA	NA	1	4.5 ± 0	4.5
Leaf	Area_y1_y2	0	NA	NA	0	NA	NA	4	3.1 ± 0.2	9.8
	Circularity_y1_y2	0	NA	NA	0	NA	NA	3	4.4 ± 0.4	11.7
	Horizontal_symmetry_y1_y2	0	NA	NA	0	NA	NA	1	10.8 ± 0	10.8
	Width_y1_y2	1	4.0 ± 0	4.0	1	4.2 ± 0	4.2	4	3.2 ± 0.3	10.0
	Indent_depth_y1_y2	0	NA	NA	0	NA	NA	1	3.2 ± 0	3.2
	Indent_width_y1_y2	0	NA	NA	0	NA	NA	1	4.8 ± 0	4.8
	Num_Indents_y1_y2	0	NA	NA	0	NA	NA	6	3.9 ± 0.7	19.8
	PC1:PC2_y1_y2	0	NA	NA	1	4.1 ± 0	4.1	5	4.2 ± 0.1	10.7
	PC1:PC3_y1_y2	0	NA	NA	0	NA	NA	7	6.2 ± 0.9	23.7
	PC1:PC4_y1_y2	0	NA	NA	1	4.1 ± 0	4.1	4	4.6 ± 1.2	12.8
	PC1_y1_y2	0	NA	NA	0	NA	NA	8	5.6 ± 0.8	21.1
	PC3:PC4_y1_y2	1	4.3 ± 0	4.3	0	NA	NA	2	3.2 ± 0.1	6.6
	PC4_y1_y2	0	NA	NA	0	NA	NA	3	4.4 ± 0.9	13.4
	Perimeter_y1_y2	0	NA	NA	0	NA	NA	2	3.1 ± 0.1	6.4
	Perimeter2:Area2_y1_y2	1	4.0	4.0	0	NA	NA	0	NA	NA
	Length:width_y1_y2	1	4.7 ± 0	4.7	0	NA	NA	7	6.9 ± 0.6	19.3
	Length_y1_y2	0	NA	NA	0	NA	NA	1	3.5 ± 0	3.5
Phenology	Bud_burst_y1_y2	0	NA	NA	1	5.6 ± 0	5.6	2	6.1 ± 0.6	6.6
	Color_y1_y2_y3	1	4.1	4.1	0	NA	NA	5	4.6 ± 0.4	14.2
	Drop_y1_y2_y3	1	4.4 ± 0	4.4	0	NA	NA	2	5.9 ± 1.2	7.9
	Green_canopy_duration_y1_y2	0	NA	NA	0	NA	NA	2	4.9 ± 0.1	6.8
	Time_serie_bud_burst_y1_y2	0	NA	NA	2	4.5 ± 0.8	7.2	2	8.6 ± 1.2	17.7
	Time_serie_color_y1_y2_y3	1	3.8 ± 0	3.8	0	NA	NA	3	4.5 ± 0.5	9.7
	Time_serie_drop_y1_y2_y3	1	4.8	4.8	0	NA	NA	3	5.2 ± 1.5	12.7

^*a*^Three major phenotypic categories (Biomass, Leaf morphology, Phenology) used for QTL analysis in this study.

^*b*^Shortcuts of trait names applied in QTL analysis. The full explanation of traits can be found in Supplementary File 2.

^*c*-*e*^Three models used for QTL analysis. Traits represent phenotypic data. *P. deltoides*, *P. nigra* and Dosage represent *P. deltoides* genotypes, *P. nigra* genotypes and Dosage states, respectively.

^*f*^Number of QTLs observed in each trait.

^*g*^Phenotypic variance explained by every observed QTL on average.

^*h*^Total phenotypic variance explained by all of the observed QTLs in one trait.

^*i*^Year 1 refers to the year 2014. Year 2 refers to the year 2015. Year 3 refers to the year 2016.

^*j*^The 2 biomass traits without year information (Diameter and Volume) are measured at a single time point corresponding to the day of harvest (December 2016).

^*k*^Biomass trait Time_serie_diameter_breast_height was the diameter at breast height, measuring as a continuous time series.

^*l*^Only the 27 traits with observed QTLs were shown here. The complete traits list is in Supplementary File 2.

**Fig. 3.**
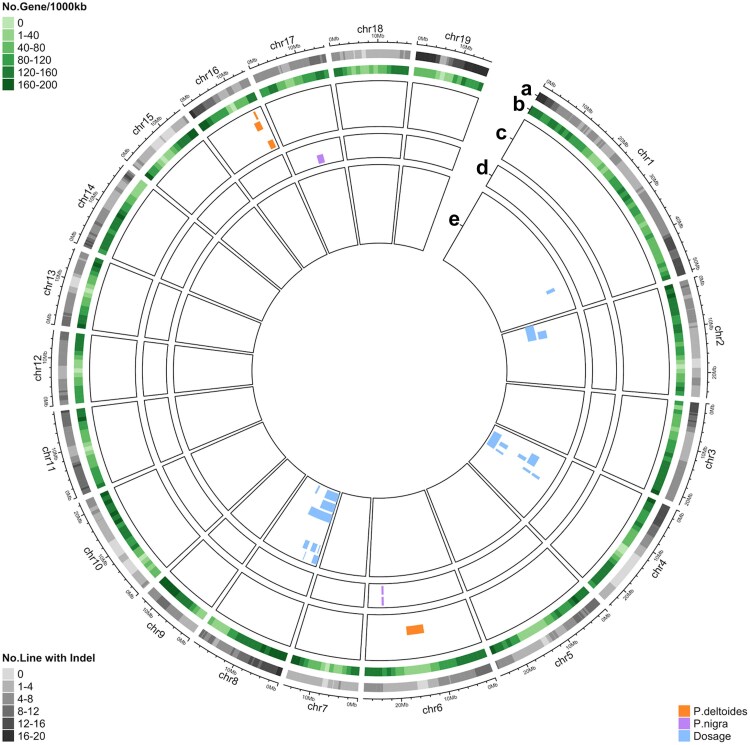
Observed QTLs for phenology traits using single models. a) Number of lines carrying indels under each bin. b) Gene density across the genome. (c to e) QTLs detected from *P. deltoides* (c)*, P. nigra* (d), and dosage (e) genotypes. The traits from outermost to innermost in each track are c) Color_y1_y2_y3, Drop_y1_y2_y3, Time_serie_color_y1_y2_y3, Time_serie_drop_y1_y2_y3; d) Bud_burst_y1_y2, Time_serie_bud_burst_y1_y2; e) Bud_burst_y1_y2, Color_y1_y2_y3, Drop_y1_y2_y3, Green_canopy_duration_y1_y2, Time_serie_bud_burst_y1_y2, Time_serie_color_y1_y2_y3, and Time_serie_drop_y1_y2_y3. Phenotypic data were obtained from previous reports, and detailed trait information is summarized in Supplementary File 1.

**Fig. 4.**
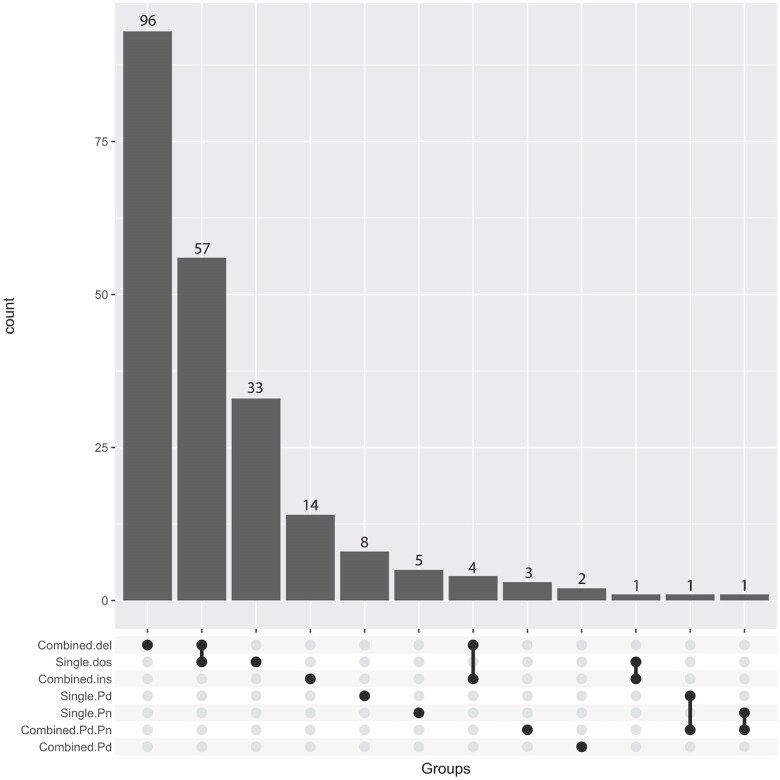
Number of QTLs detected from single and combined models. Single.dos: QTLs from the single model Trait ∼ Dosage. Single.Pd: QTLs from the single model Trait ∼ *P. deltoides* haplotypes. Single.Pn: QTLs from the single model Trait ∼ *P. nigra* haplotypes. Combined.del: QTLs associated with a deletion. Combined.ins: QTLs associated with an insertion. Combined.Pd: QTLs associated with the *P. deltoides* haplotypes. Combined.Pd.Pn: QTLs associated with an insertion and the *P. deltoides* and *P. nigra* haplotypes.

Overall comparison of the number of QTLs detected using the three single models reveals that dosage variation has the most pronounced impact on phenotypic variation (Fig. 3, Supplementary Figs. 5 and 6). Interestingly, QTLs observed from the 3 single models did not overlap with each other (Fig. 4), indicating that natural variation in the two parental species, *P. deltoides* and *P. nigra*, and dosage variation may influence these traits independently.

To investigate to what extent indels can affect the identification of allelic QTL results, we selected the 157 lines from this F1 population that did not carry any indels and tested the identification of allelic QTL on this subset. In total, 1 and 8 allelic QTLs were identified from the *P. deltoides* and *P. nigra* parents, respectively (Supplementary Table 1 and Supplementary File 4). Interestingly, there were no common allelic QTLs between the subset population (157 lines) and the full population (343 lines). A subset of both sets of allelic QTLs overlapped with previously published QTLs. For example, for the allelic QTLs in the full population, *P. nigra* QTLs on chromosomes 6 and 17 for phenology-related traits (bud burst) were consistent with previously reported allelic QTLs (Frewen *et al.* 2000; Rohde *et al.* 2011; Fabbrini *et al.* 2012). For the allelic QTLs in the subset population, *P. nigra* QTLs on chromosome 3 for phenology-related traits (bud burst) and leaf shape were consistent with reported QTLs in *Populus* (Rohde *et al.* 2011; Xia *et al.* 2018). These results suggest that the identification of allelic QTL in the full population is significantly affected by the presence of the large-scale indels, which could completely mask the effect of some or all of the allelic QTLs when present.

**Fig. 5.**
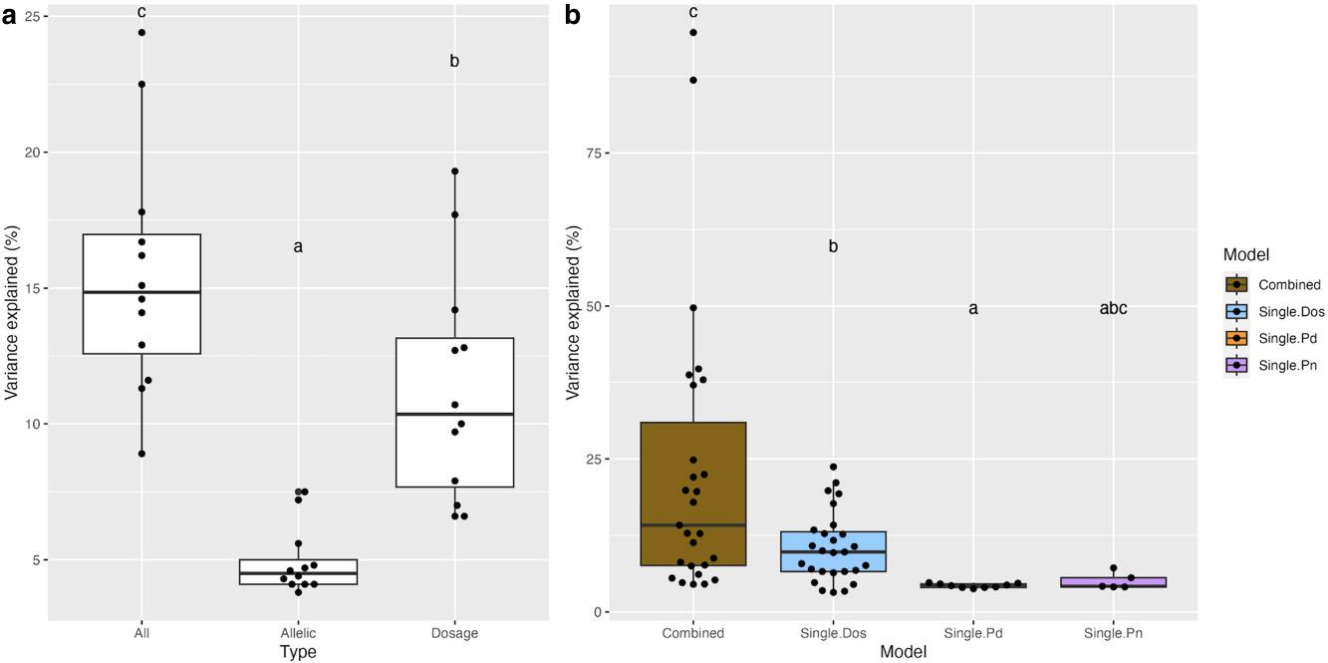
Phenotypic variance explained using the single and combined models. a) Phenotypic variance explained by allelic and dosage variation using single models. 12 traits were selected for the observation of both allelic and dosage QTLs. On the *x*-axis, *All* represents the variance explained by all QTLs identified using the three single models. *Allelic* represents the variance explained by the collection of QTLs from *P. deltoides* and *P. nigra* haplotypes. *Dosage* represents the variance explained by dosage variation. b) Comparison of the percentage of phenotypic variance explained by the single and combined models. On the *x*-axis, *Combined* represents the variance explained by QTLs observed from the combined model. *Single.Dos*, *Single.Pd* and *Single.Pn* represent QTLs identified from three single models associated with dosage variation, *P. deltoides* haplotypes, and *P. nigra* haplotypes, respectively. Statistical significance was calculated through pairwise permutation tests (*P*-value < 0.05).

Coming back to the full population, allelic variation and dosage variation explained 4.94 and 11.27% phenotypic variance, respectively (Fig. 5a). To investigate whether combining the effects of natural allelic variation and induced dosage variation can explain a larger percentage of the observed phenotypic variance, we used a multivariate model to detect allelic and dosage QTLs simultaneously. We first selected 12 traits for which both allelic and dosage variation were associated with detected QTLs (Supplementary File 5). Integration of QTLs from the three single models explained 15.51% of the observed phenotypic variance in these 12 traits. This percentage was significantly higher than the percentage of variance explained by either allelic variation alone (Tukey's test, *P* < 0.001) or dosage variation alone (Tukey's test, *P* = 0.019; Fig. 5a and Supplementary File 6).

To investigate the molecular mechanism underlying the detected QTLs, we identified the genes located within the observed QTL regions and examined their differential expression levels based on the leaf transcriptomic data from our previous study (Bastiaanse *et al.* 2020a) (Supplementary File 7). GO enrichment analysis indicated that differentially expressed genes (DEGs) associated with allelic QTLs were suggestively enriched with translation (0.05 < *P*-value < 0.1; Supplementary Fig. 7), while DEGs associated with dosage QTLs were significantly enriched with stress response processes (Bastiaanse *et al.* 2020a).

### A combined univariate model helps refine our understanding of trait regulation

**Fig. 6.**
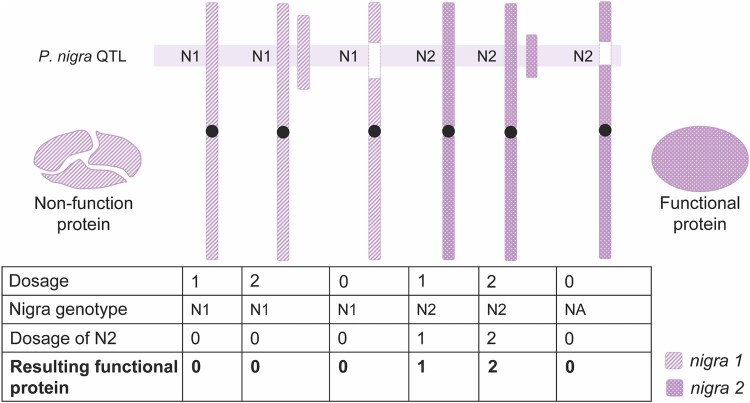
Representative diagram of possible interplay between *P. nigra* haplotypes and dosage variation. N1 (*P. nigra 1*) encodes a non-functional protein, while N2 (*P. nigra 2*) encodes a functional protein. Copy number changes on N1 have no effect on phenotypes, while copy number changes on N2 result in dramatic differences on phenotypic outcomes. Chromosomes inherited from *P. deltoides* (not shown) are always present in one copy.

Allelic and dosage variation effects may also interact with each other. For example, dosage effects are expected to be different if the causal gene also carries a loss-of-function allele (Fig. 6). To better understand the interaction between the effects of natural allelic variation and induced dosage variation, we combined the information from the three variation types and assigned each combined genotype to a unique state. For example, D1.N1.1 on marker 1 represents the individuals with *P. deltoides* haplotype *1*, *P. nigra* haplotype *1*, and 1 *P. nigra* copy for marker 1. In this model, all individuals fit into one of 10 possible states, and we can incorporate these integrated genotypic states into a univariate model, such as Trait ∼ States (Supplementary File 2). Next, pairwise comparisons can be performed between groups in the different genotype states using linear regression. Loci exhibit significant phenotypic differences through pairwise comparison and were assigned as QTLs. We categorized these QTLs into 6 groups (deletion, insertion, deletion + insertion, *P. deltoides*, *P. nigra*, and *P.deltoides + P. nigra*), according to the phenotypic differences between compared genotypic states (see Materials and Methods).

**Fig. 7.**
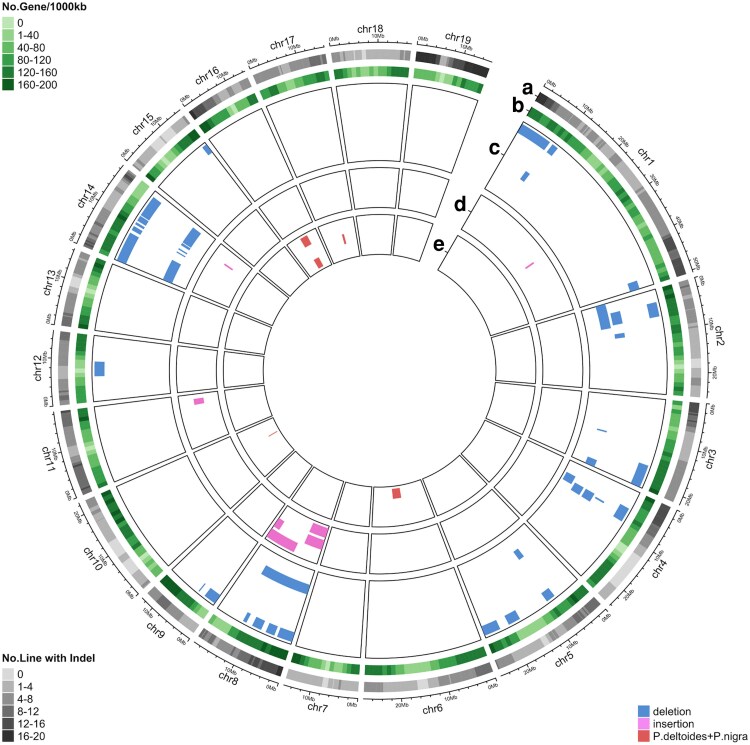
Observed QTLs for the phenology traits using the combined model. a) Number of lines carrying indels under each bin. b) Gene density across the genome. (c to e) QTLs detected based on variation in deletion (c), insertion (d), and *P.deltoides + P.nigra* haplotypes (e). The traits from outermost to innermost in each track are c) Bud_burst_y1_y2, Color_y1_y2_y3, Drop_y1_y2_y3, Green_canopy_duration_y1_y2, Time_serie_bud_burst_y1_y2, Time_serie_drop_y1_y2_y3; d) Color_y1_y2_y3, Drop_y1_y2_y3, Time_serie_drop_y1_y2_y3; e) Color_y1_y2_y3, and Time_serie_bud_burst_y1_y2, Time_serie_color_y1_y2_y3.

In total, we observed 163 QTLs from the combined model that belonged to 4 different groups [deletion, insertion, *P. deltoides*, and *P.deltoides + P. nigra* (Table 2 and Supplementary File 4)]. Among these 4 groups, most QTLs were associated with deletions (Fig. 4). This result is consistent with expectation from single models, since dosage variation was associated with QTLs much more often than allelic differences (Fig. 4). These findings are also illustrated in the Circos plots, where deletions (Fig. 7c, Supplementary Figs. 8c and 9c) are associated with most QTLs, followed by insertions (Fig. 7d, Supplementary Figs. 8d and 9d), and allelic variation (Fig. 7e, Supplementary Figs. 8e and 9e). These observations confirmed that dosage variation drives phenotypic variation for most traits in our population, while variation in parental haplotype did not strongly modulate the effects of dosage variation.

**Table 2.** QTLs obtained using the combined model.

Phenotype (# of traits)	Groups*^a^*	Total # of QTL	# of traits with QTL	Variance explained by single QTL (µ ± σ) (%)	Variance explained by all QTLs of a trait (µ ± σ) (%)
Biomass (9)	deletion	14	6	7.6 ± 6.7	12.3 ± 5.6
Leaf (22)	deletion	84	12	6.4 ± 4.2	27.1 ± 30.2
	insertion	12	5	13.0 ± 12.0	
	*P. deltoides*	2	2	6.9 ± 1.1	
Phenology (7)	deletion	39	6	5.8 ± 4.4	24.5 ± 14.1
	insertion	7	3	10.3 ± 7.0	
	*P. deltoides + P. nigra*	5	3	5.0 ± 1.1	

^*a*^The observed QTLs were categorized into groups based on their origin: deletion, insertion, deletion + insertion, *P. deltoides*, *P. nigra*, *P. deltoides + P. nigra.* This table only shows groups for which QTLs were identified.

The combined model detected only a few instances where the QTLs observed by different genotypes overlapped (Fig. 4). These QTLs were associated with leaf shape and localized on chromosome 17 (Supplementary Fig. 9), where they were associated with both deletions and insertions. This result is consistent with the outcome from the single model analysis, indicating that dosage and allelic variation may independently affect the examined traits.

**Fig. 8.**
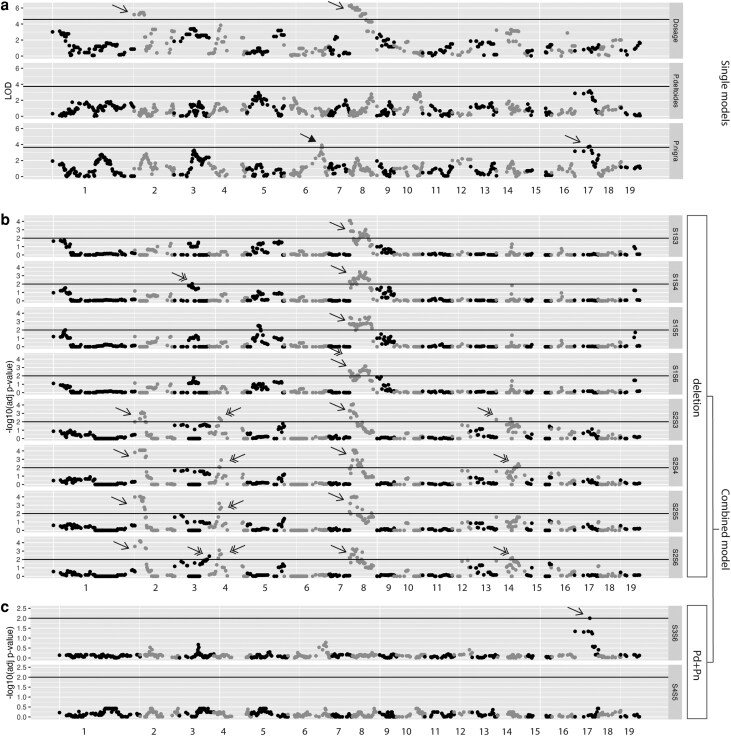
Examples of QTLs identified using the single and combined models on the phenology-related trait Time_serie_bud_burst_y1_y2. Each dot represents a genetic marker. The X-axis indicates genomic positions. The *y*-axis indicates the LOD scores (a) or adjusted *P*-value with negative log10 fold (b, c). Dots above the horizontal lines were selected as QTLs. These QTLs were categorized as observed only in the single models (triangle arrow), only in the combined model (double arrows) and in both single and combined models (single arrows). a) LOD scores of genetic markers from three single models. Top: Trait ∼ Dosage; Middle: Trait ∼ *P. deltoides* haplotype; Bottom: Trait ∼ *P. nigra* haplotype. (b, c) Combined model categorized QTLs into 6 groups based on their origin. Deletion (b) and *P. deltoide* + *P. nigra* (Pd + Pn) (c) groups are shown here because QTLs were identified in these comparisons. b) Each plot represents one pairwise comparison between two genotype states for which genotypes are equal but dosage varies. c) Each plot represents one pairwise comparison between two genotype states for which dosage is equal but the *P. deltoides* and *P. nigra* haplotypes vary.

Finally, we investigated the percentage of phenotypic variance explained by the QTLs identified using the combined model. To calculate phenotypic variance for each trait, QTLs belonging to the same trait were merged. Merged QTLs explained on average 23.2% of the phenotypic variance, which is significantly higher than the variance explained from dosage variation only (on average 10.6%) or *P. deltoides* haplotype variation (on average of 4.3%) (permutation test, *P*-value < 0.05), and is suggestively higher than only *P. nigra* haplotypes (on average of 5.1%; permutation test, *P*-value < 0.1) (Fig. 5b and Supplementary File 6). Meanwhile, we observed that the integration of QTLs from all three single models explained a smaller percentage of the phenotypic variance than the QTLs from the combined model (12.2% vs 23.2%; permutation test, *P*-value < 0.05). Presumably, the increase originates from the QTLs identified using the combined model but not identified using the single models. Some of these QTLs were shown to be suggestive (0.05 < *P*-value < 0.1) when using the single models (Fig. 8a, chromosomes 3, 4), while others were not identified at all using the single model (Fig. 7, chromosome 14). These findings confirm the advantage of using a combined model approach.

## Discussion

Identifying candidate genes underlying a target trait is a crucial step toward understanding the mechanisms affecting the trait, and for applying this knowledge to plant breeding. Quantitative trait loci (QTL) analysis, which typically correlates SNP to traits or phenotype-associated features such as gene expression and RNA alternative splicing (Brem *et al.* 2002; Li *et al.* 2016), is an efficient approach for this endeavor. Besides SNPs, other genetic features such as dosage variation (Bastiaanse *et al.* 2019, 2020a; Rodriguez-Zaccaro *et al.* 2021) can affect traits of interest. A unique *Populus* population, which carries natural allelic variation and induced dosage variation was previously established (Henry *et al.* 2015b). Previous analysis demonstrated few point mutations and small indels in this population (Henry *et al.* 2015b), indicating that preexisting SNPs and induced large-scale indels are the major sources of genetic variation in this population and presumably drive the observed phenotypic variation. In our study, we aimed to investigate the effects of natural allelic variation and induced dosage variation on quantitative traits. In general, our results indicate no overlap between QTLs from natural and dosage variation in our system.

A single model approach was used to describe the correlation between each source of variation and target traits. *P. deltoides* and *P. nigra* genotypic information allowed for the identification of QTLs between different haplotypes within each parental species. Compared with previous QTL analysis in other *Populus* cross populations (Rae *et al.* 2009; Rohde *et al.* 2011; Fabbrini *et al.* 2012), our study found fewer allelic QTLs. As demonstrated by our research identifying QTLs in the subset of trees that do not carry large indels, this may be because the presence of many large indels may mask the observation of QTLs associated with natural allelic variation. For example, dosage-sensitive genes can play the trans-regulatory factors and affect large numbers of genes across the genome (Bastiaanse *et al.* 2020a). Interestingly, we found no overlap between the *P. deltoides* QTLs and the *P. nigra* QTLs. A previous study (Rohde *et al*. 2011) also reported no overlap between *P. deltoides* and *P. nigra* QTLs when the two species were used as the two parents of the same population (*P. deltoides × P. nigra*), which is consistent with our results. However, in the same study, shared QTLs were observed if *P. deltoides* and *P. nigra* were used in different crosses (Rohde *et al.* 2011). This might be because, if both *P. deltoides* and *P. nigra* carry genetic variation at the same location and both parental genotypes affect the trait, the source of phenotypic variation is more difficult to identify. Instead, when they are crossed with other *Populus* species, which do not carry variations that affect the trait, QTLs can be detected. With the current data, it is difficult to determine if the pathways that control these three phenotypic categories—biomass, leaf morphology, and phenology—are similar or not.

Dosage variation was induced by γ irradiation of *P. nigra* pollen and all resulting indels are located on the *P. nigra* chromosomes (Henry *et al.* 2015b). Therefore, we expected to observe some overlap between *P. nigra* allelic QTLs and dosage QTLs. For example, if the *P. nigra* QTL is associated with alleles affecting gene expression levels, then dosage and allelic variation would have similar effects, with decreased protein level to 0 in the case of deletion or increased levels to two-folds in the case of an insertion. According to this model, both *P. nigra* QTL and dosage QTL act through dosage-dependent regulation of the target trait. The dosage-dependent behavior is consistent with additivity and has been described as the basis for quantitative variation (Lukens and Doebley 1999; Frary *et al.* 2000).

Surprisingly, dosage QTLs and allelic QTLs do not overlap (Fig. 4). There can be multiple reasons for this outcome, depending on the mechanisms underlying the QTL at hand. For loci that display only allelic QTL, the impact of 1× to 2× constitutive dosage variation might be insufficient to affect protein function, whereas allelic variation could potentially affect gene function through more drastic modifications, such as significantly altering the expression pattern, or directly affecting the protein function if there are changes in the amino acid sequence. It is also possible that dosage variation at those loci was absent or too infrequent in the indel population for the detection of a dosage QTL effect. Indeed, over 50% of the *P. nigra* loci are connected to fewer than 5 indels (Henry *et al.* 2015b), limiting the statistical power of our dosage QTL analysis. Finally, gene dosage compensation is another possible explanation, in which the structural gene dosage effect is canceled by an inverse regulatory effect, exerted either within the same locus or from an unlinked region (Birchler *et al.* 1990; Birchler and Veitia 2012). The combination of these two opposite effects would result in no significant change of gene expression. Conversely, for loci for which only dosage QTLs were detected, it is possible that natural allelic variation is not present at these loci, or that it has too subtle an impact to affect the associated phenotype. The gene balance hypothesis can explain the success in detecting dosage QTLs and the failure of detecting allelic QTLs in the case of genes encoding proteins that are part of multisubunit complexes. According to this hypothesis, traits regulated by multisubunit complexes are particularly sensitive to dosage. Copy number variations involving the genes encoding these subunits can perturb their stoichiometry, leading to a dramatic alteration in the protein complex function and, ultimately, impacting the connected traits (Birchler and Veitia 2012). On the other hand, sequence variation with subtle effects would be difficult to identify (Birchler and Veitia 2021).

Integration of QTLs from dosage and allelic variation, compared to either allelic QTLs or dosage QTLs alone, significantly improved the percentage of variance explained (Fig. 5a). These results suggest that a large proportion of the phenotypic variation was caused by the induced large-scale indels, but not all of it. Some of the phenotypic variation is caused by natural allelic variation, and taking both the allelic and dosage variation into account improves phenotypic prediction. However, the integration of all identified QTLs from the single models explained, on average, only 12.2% of the observed phenotypic variance, indicating that the majority of the variance remains unexplained. This could be due to the interaction between allelic and dosage variation. For example, dosage effects are expected to be allele-sensitive if the responsible gene is heterozygous for a null allele (Fig. 6). As a result, single models focusing solely on natural allelic variation or induced dosage variation are not able to identify these interactive effects.

We next developed a combined model including all variation types. We categorized the QTLs into 6 groups based on the following types of variation: deletion, insertion, deletion + insertion, *P. deltoides* haplotypes, *P. nigra* haplotypes, and *P. deltoides + P. nigra* haplotypes. Most QTLs were associated with dosage-related groups, with deletions being the most common cause, followed by insertions. QTLs associated with allelic variation (*P. deltoides*, *P. nigra*, and *P. deltoides + P. nigra* haplotypes) were the least common. Most QTLs were observed within dosage-related groups. Possibly, this is because dosage variants were newly induced and have not experienced selection. There was no overlap between allelic and dosage QTLs, which is consistent with the results obtained using the single models.

Taken together, we investigated the contribution of natural allelic variation and induced dosage variation in F1 *Populus* hybrids on quantitative traits. We found no overlap between allelic and dosage variation QTLs, suggesting that the naturally occurring sequence polymorphisms and the induced structural variation influence the traits under different constraints and through different mechanisms. Integrating the QTLs from allelic and dosage variation significantly increased the proportion of phenotypic explained variance compared to considering only allelic or dosage QTLs. A new method was designed to include all types of variation simultaneously for QTL analysis, and it was applied to investigate the interaction between allelic and dosage variation in detail. This novel approach significantly increased the explained proportion of phenotypic variance and revealed that genomic fragment deletion had the most pronounced effect on traits. The future direction would be to identify responsible genes within the QTL intervals as a next step toward helping the development of *Populus* clones with commercial benefits.

## Supplementary Material

jkae218_Supplementary_Data

## Data Availability

The sequences reported in this paper were previously deposited (Henry *et al.* 2015b; Bastiaanse *et al.* 2020a) and can be found in the National Center for Biotechnology Information BioProject Database (BioProject ID: PRJNA241273 and PRJNA646735) (Henry *et al*. 2015a; Bastiaanse *et al*. 2020b). Supplemental material available at G3 online.
