# Supplementary material for: Induced and natural variation affect traits independently in hybrid Populus
Source: G3 (Bethesda). 2024 Sep 12;14(11):jkae218. doi: 10.1093/g3journal/jkae218 (PMC11540314; doi:10.1093/g3journal/jkae218)
Supplement: jkae218_Supplementary_Data [file jkae218_supplementary_data.zip › Supplemental_Figures_and_Table_G3-2024-405349.pdf]

Supplementary Figures and Table

Table S1. List of allelic QTLs obtained from 157 nonindel lines.

Category	Traits	Trait ~ P.deltoides			Trait ~ P.nigra		
		# of QTL	% explained by single QTL (μ ± σ)	% explained by all QTLs	# of QTL	% explained by single QTL (μ ± σ)	% explained by all QTLs
Leaf	Circularity_y1_y2	0	NA	NA	3	11.5 ± 1	28.3
	Length_y1_y2	1	9.1	9.1	0	NA	NA
	Horizontal_symmetry_y1_y2	0	NA	NA	1	8.8	8.8
	PC1:PC2_y1_y2	0	NA	NA	2	11.8 ± 3.2	18.4
	PC2:PC3_y1_y2	0	NA	NA	1	9.7	9.7
Phenology	Bud_burst_y1_y2	0	NA	NA	1	12	12

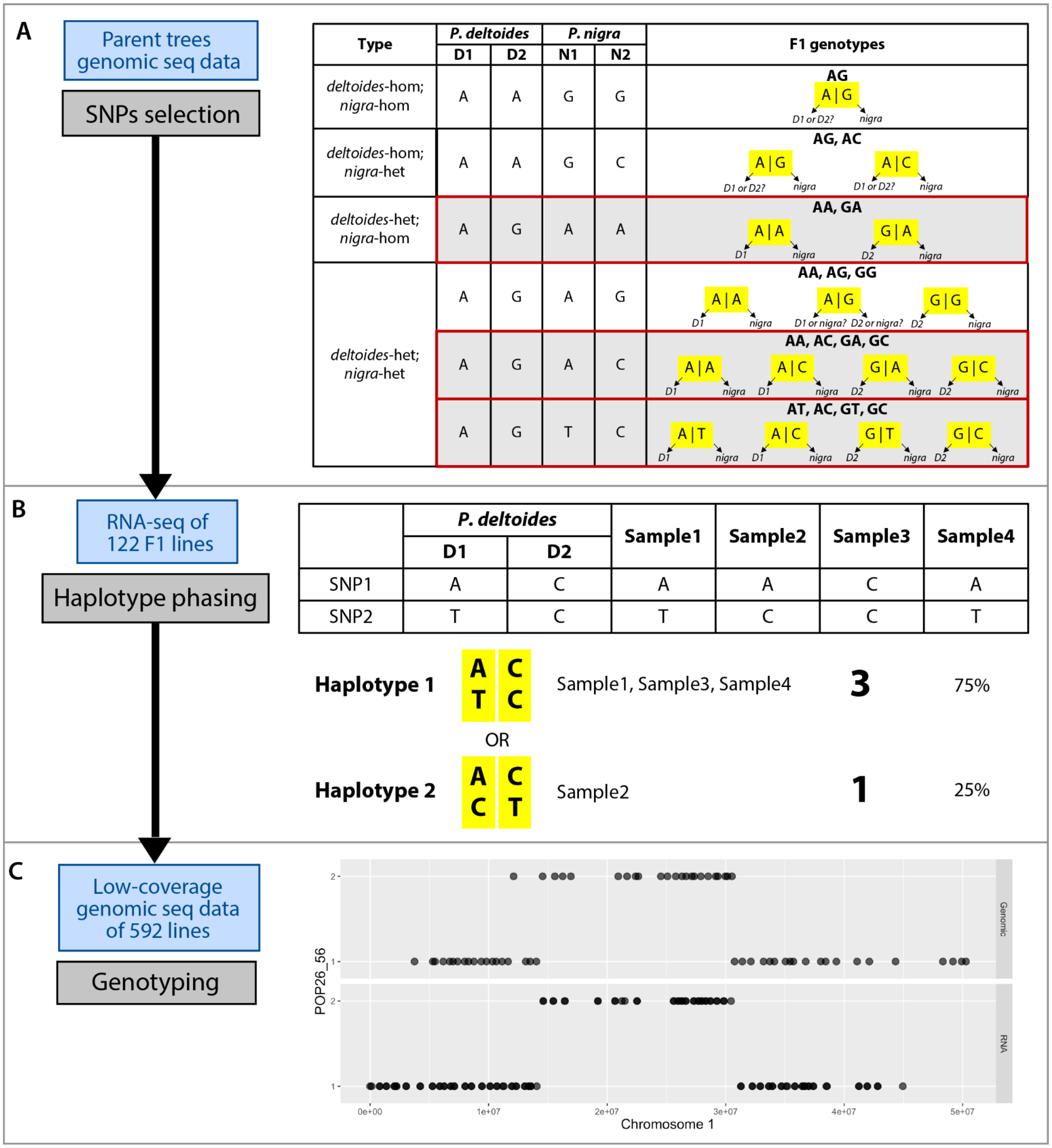

Figure S1. F1 genotyping approach. For each panel on the left, the blue box represents the data used, and the black box represents the pipeline step. The diagrams on the right side provide more details for each step. (A) Selection of SNPs that can distinguish between the two parental haplotypes (‘D1’ and ‘D2’ for *P. deltoides* haplotype 1 and 2, ‘N1’ and ‘N2’ for *P. nigra* haplotype 1 and 2). The table on the right lists all possible F1 genotypes.The rows highlighted in gray background and red frame indicate the positions selected for *P. deltoides* haplotyping. (B) Haplotype phasing using high-coverage RNA-seq data. The panel on the right exemplifies how we determined parental phasing between two adjacent SNP using only 4 RNA-seq samples. In practice, genotype information from 122 RNA-seq lines were applied at this step and the threshold for an acceptable haplotype combination was set to 90% (110 out of 122).

(C) Extrapolate F1 genotypes by applying the phased haplotypes information to the low-coverage sequencing data. The panel on the right shows a comparison of the *P. deltoides* haplotypes obtained from low-coverage sequencing data (top plot) and from RNA-seq data (bottom top) for chromosome 1.

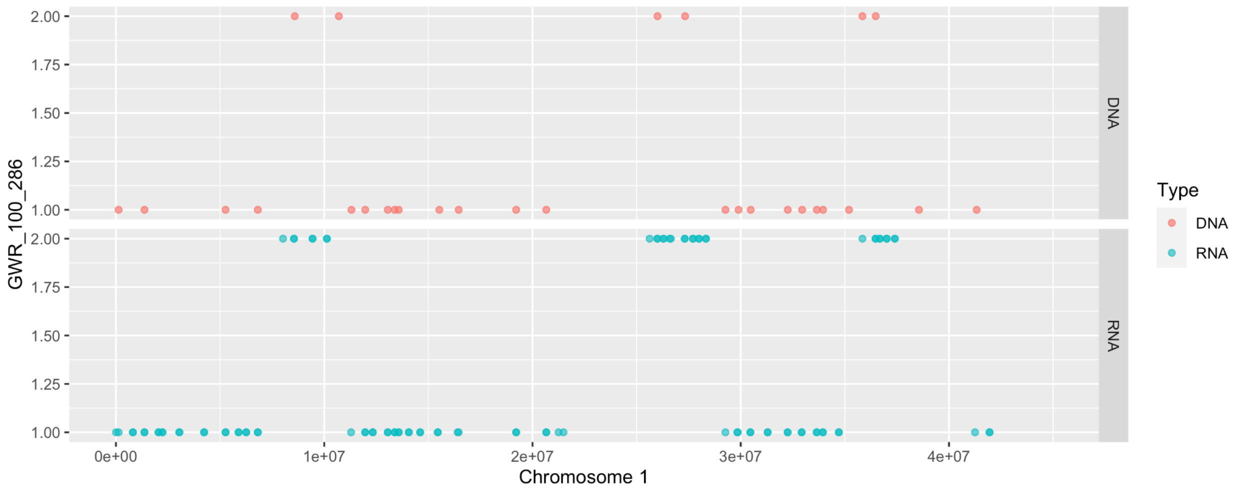

Figure S2. Comparison of genomic and transcriptomic genotypes of *P. nigra* haplotypes on chromosome 1 of the F1 lines GWR_100_286. The x-axis represents the genomic positions on chromosome 1. The y-axis represents the two haplotypes, labeled 1 and 2 here. The top panel shows genotypes generated from low-coverage genome sequencing. The bottom panel shows genotypes generated from RNA-seq data.

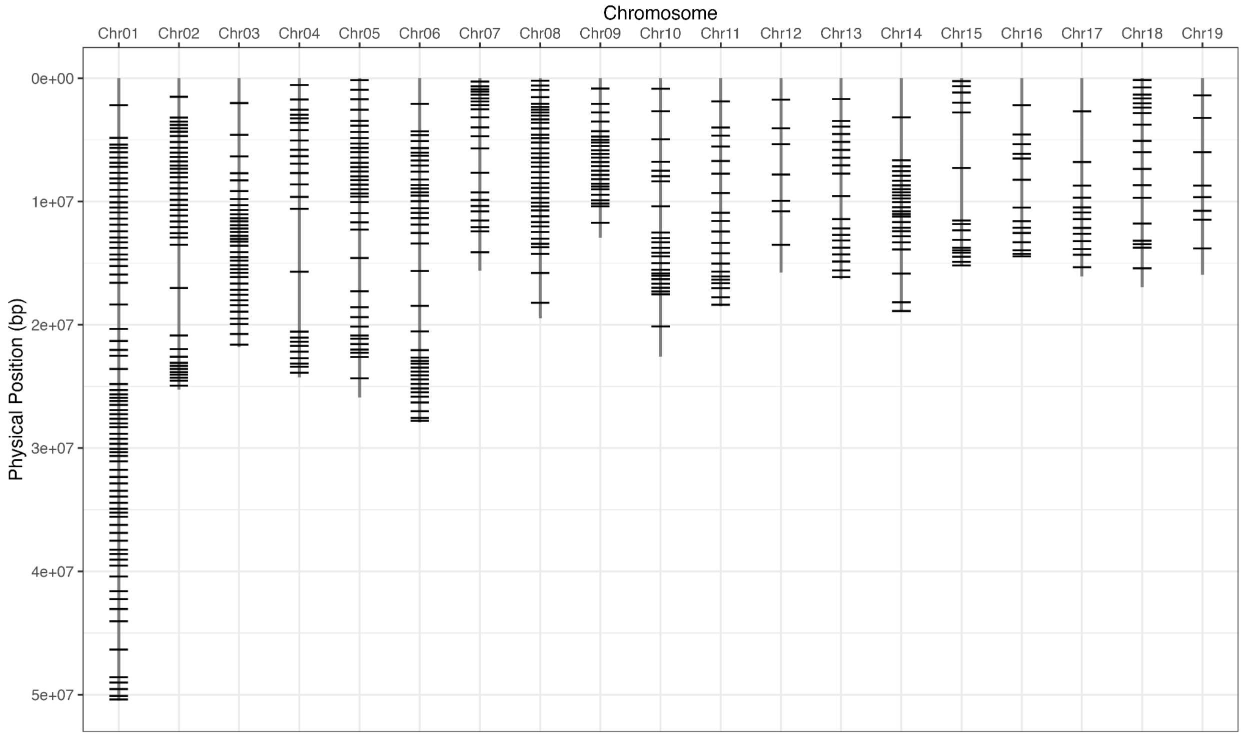

Figure S3. The physical map of 507 markers used in multi-genotype QTL analysis.

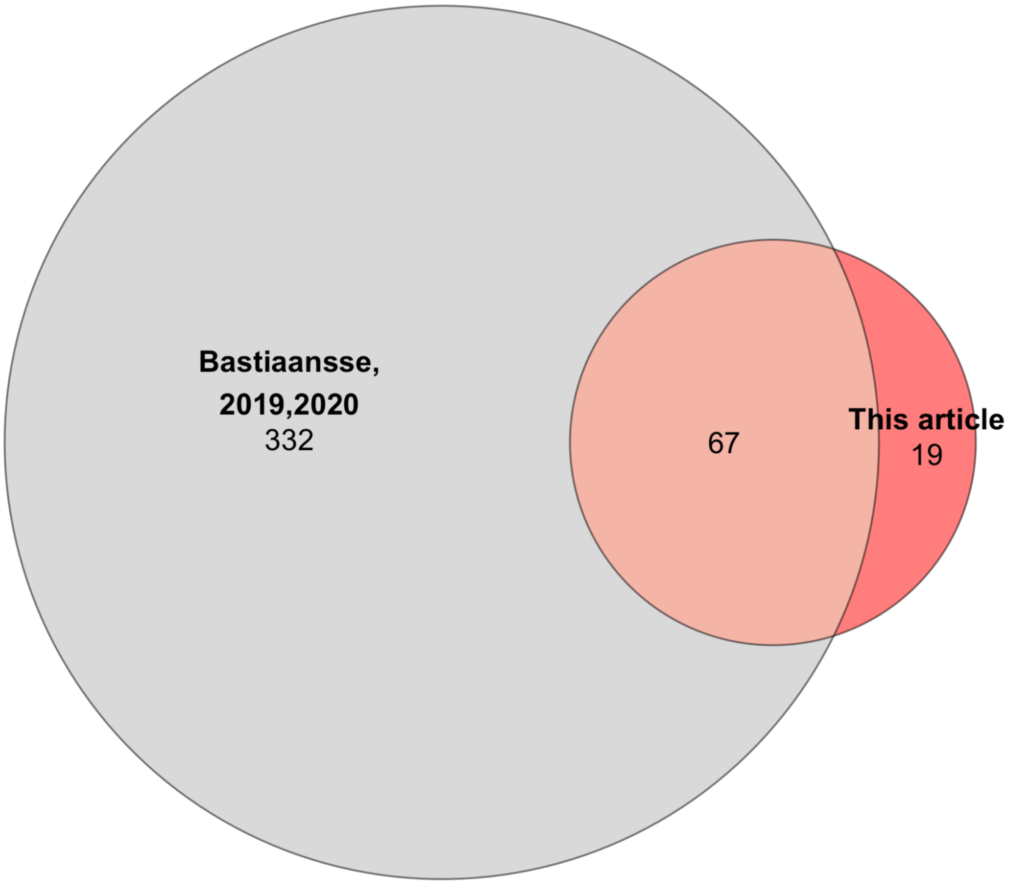

Figure S4. Comparison of dosage QTLs observed in this article (with 343 F1 hybrids) and previous studies (with 592 F1 hybrids). Left circle represents dosage QTLs observed in previous studies (Bastiaanse *et al.* 2019, 2020). The right circle represents the dosage QTLs observed through the QTL model Trait ~ Dosage in this study. The overlap region represents the common dosage QTLs generated from the two analyses. Numbers in the circles indicate QTL counts.

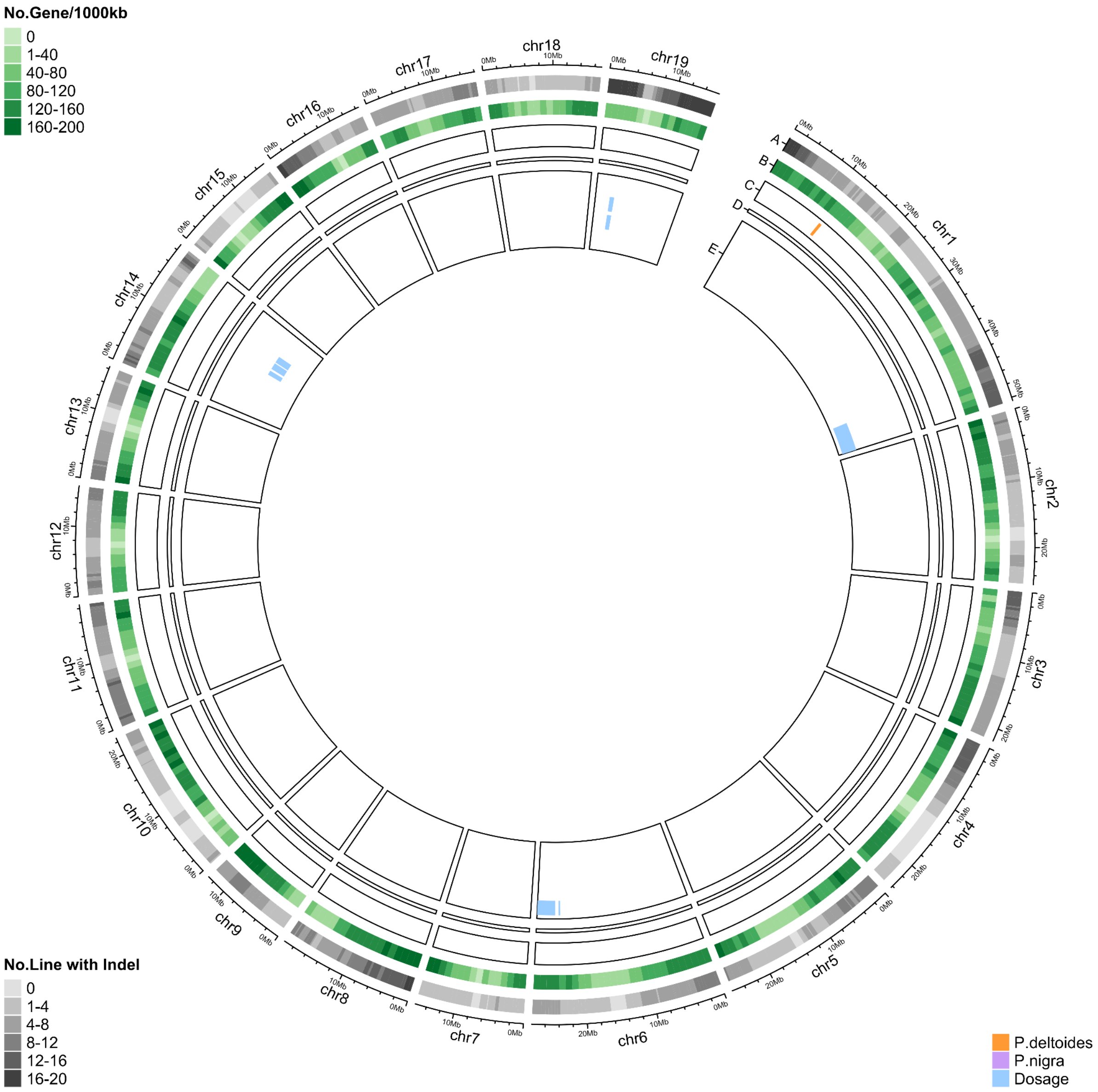

Figure S5. Observed QTLs for biomass-related traits with the single models. (A) Number of lines carrying indels under each bin. (B) Gene density across the genome. (C-E) QTLs detected from *P. deltoides* (C), *P. nigra* (D) and dosage (E) genotypes. The traits from outermost to innermost in each track are: (C) Coppicing_y1; (D) No QTL observed associated with *P. nigra* haplotypes; (E) Coppicing_y1, Diameter_base, Time_serie_diameter_base_height, Volume.

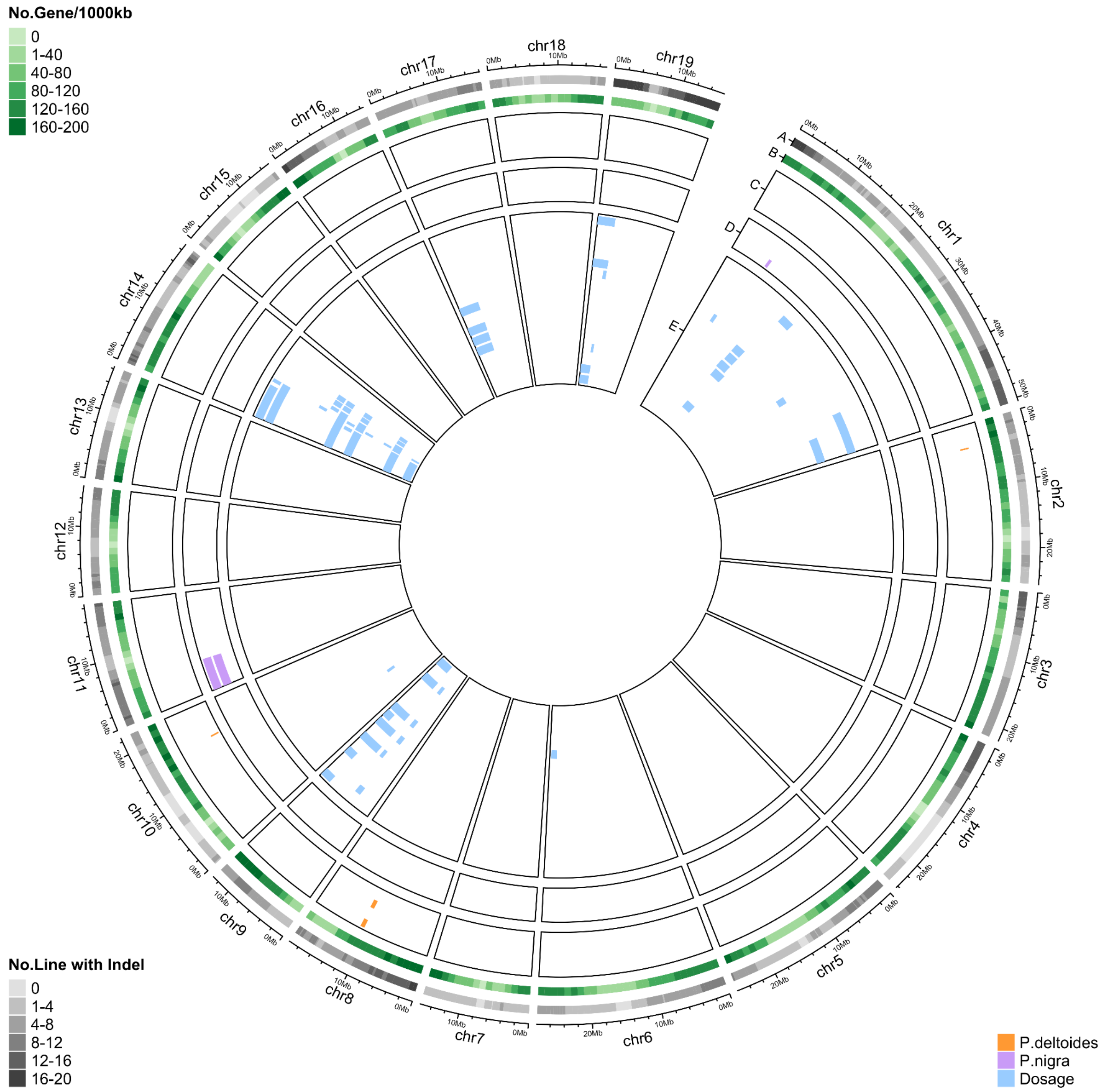

Figure S6. Observed QTLs for leaf morphology traits, using the single models. (A) Number of lines carrying indels under each bin. The bins were defined by the boundaries of indels which are tiled on the genome. (B) Gene density across the genome. (C-E) QTLs detected based on variation in *P. deltoides* haplotypes (C), *P. nigra* haplotypes (D) and dosage (E). The traits from outermost to innermost in each track are: (C) Width_y1_y2, PC3:PC4_y1_y2, Perimeter2:Area2_y1_y2, Length:width_y1_y2; (D) Width_y1_y2, PC1:PC2_y1_y2, PC1:PC4_y1_y2; (E) Area_y1_y2, Circularity_y1_y2, Horizontal_symmetry_y1_y2, Width_y1_y2, Indent_depth_y1_y2, Indent_width_y1_y2, Num_Indents_y1_y2, PC1:PC2_y1_y2, PC1:PC3_y1_y2, PC1:PC4_y1_y2, PC1_y1_y2, PC3:PC4_y1_y2, PC4_y1_y2, Perimeter_y1_y2, Length:width_y1_y2, Length_y1_y2.

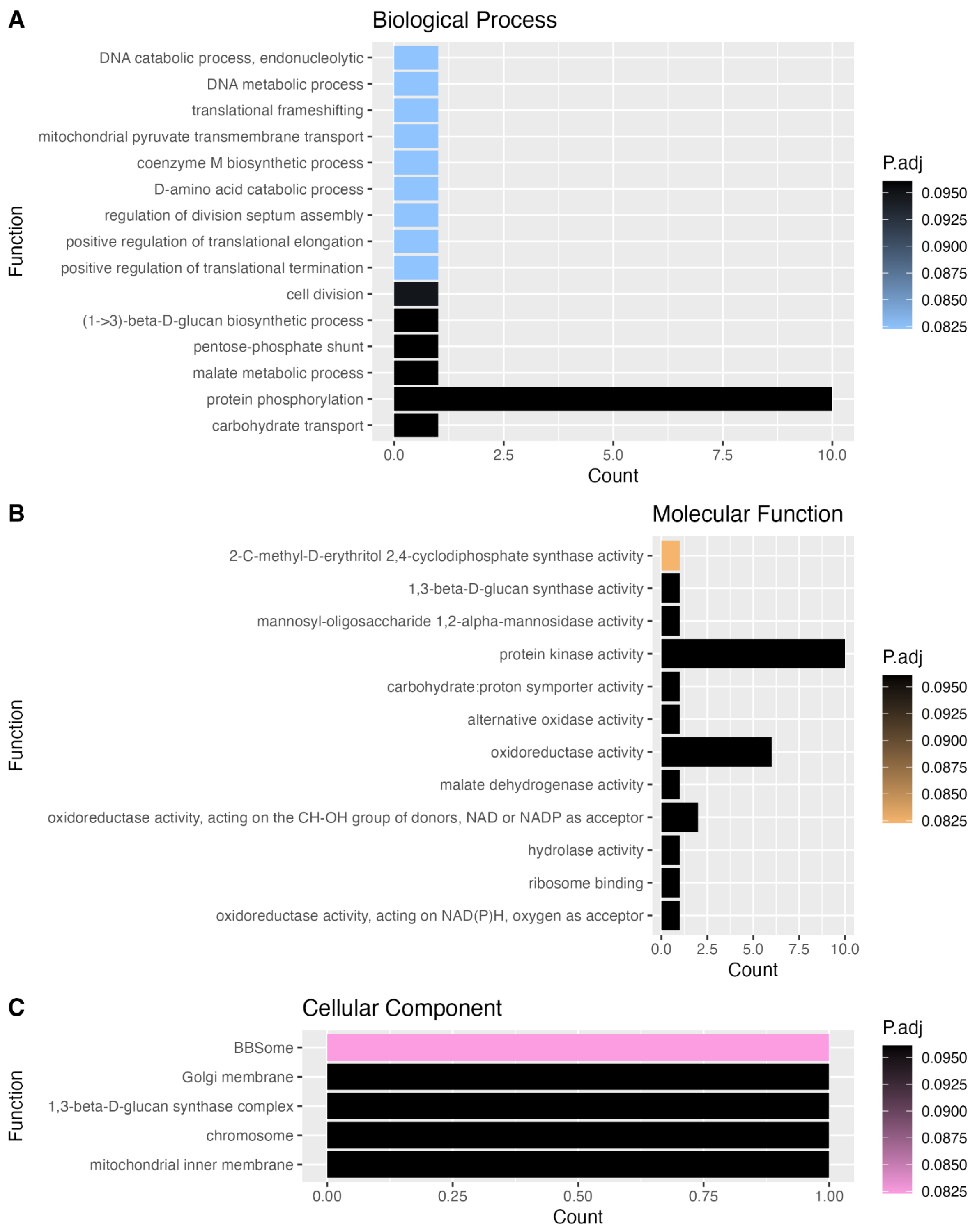

Figure S7. GO enrichment analysis for differentially expressed genes in allelic QTLs. GO terms which are suggestively enriched in allelic QTLs are shown (p-value < 0.1), and are grouped into Biological Process (A), Molecular Function (B) and Cellular Component (C). x-axis represents the gene count for enriched GO terms, y-axis lists the function of every GO term. Lighter color indicates smaller p-value.

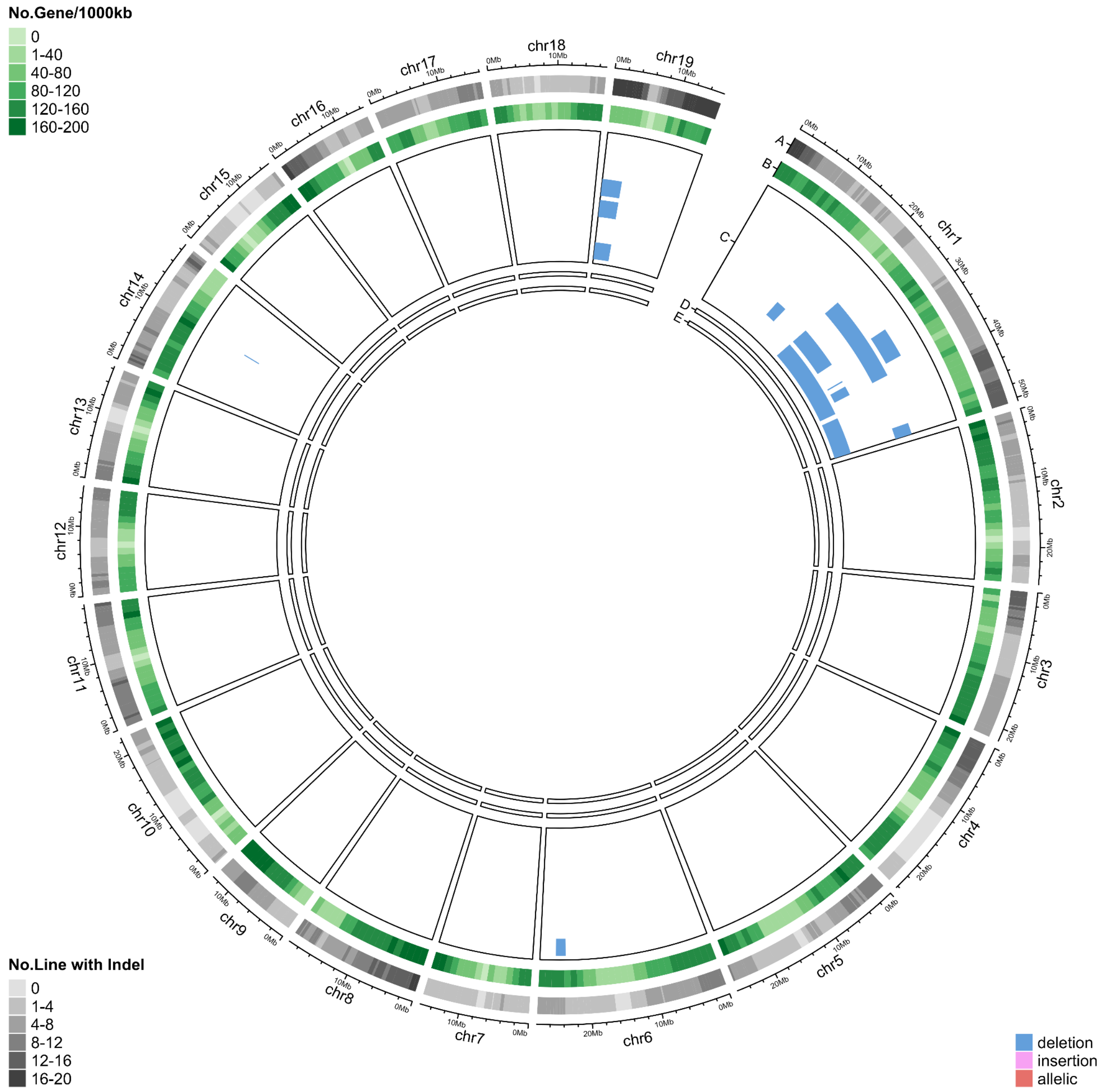

Figure S8. Observed QTLs for biomass traits using the combined model. (A) Number of lines carrying indels under each bin. (B) Gene density across the genome. (C-E) QTLs detected based on variation in deletion (C), insertion (D) and allelic (*P. deltoides* and/or *P. nigra* haplotypes change) (E). The traits from outermost to innermost in each track are: (C) Coppicing_y1, Height, Time_serie_diameter_base, Time_serie_diameter_base_height, Time_serie_height, Time_serie_volume.

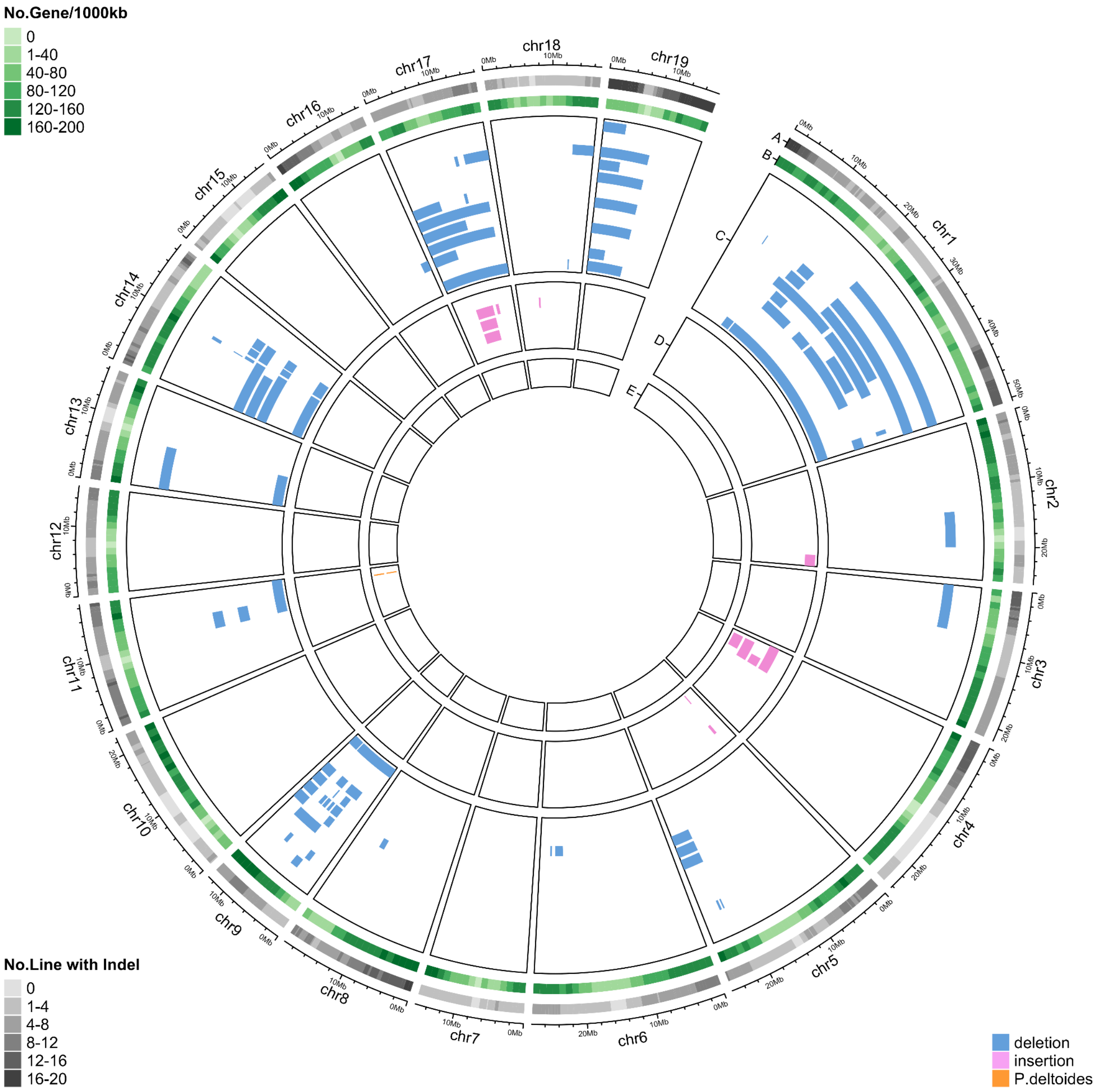

Figure S9. Observed QTLs for leaf morphology traits using the combined model. (A) Number of lines carrying indels under each bin. The bins were defined by the boundaries of indels which are tiled on the genome. (B) Gene density across the genome. (C-E) QTLs detected based on variation in deletion (C), insertion (D) and *P.deltoides* haplotypes (E). The traits from outermost to innermost in each track are: (C) Area_y1_y2, Circularity_y1_y2, Horizontal_symmetry_y1_y2, Width_y1_y2, Num_Indents_y1_y2, PC1:PC2_y1_y2, PC1:PC3_y1_y2, PC1:PC4_y1_y2, PC1_y1_y2, PC4_y1_y2, Length:width_y1_y2; (D) Horizontal_symmetry_y1_y2, PC1:PC3_y1_y2, PC1:PC4_y1_y2,, PC1_y1_y2, Length:width_y1_y2; (E) Indent_depth_y1_y2, Perimeter2:Area2_y1_y2.
