# Supplementary material for: Induced and natural variation affect traits independently in hybrid Populus
Source: G3 (Bethesda). 2024 Sep 12;14(11):jkae218. doi: 10.1093/g3journal/jkae218 (PMC11540314; doi:10.1093/g3journal/jkae218)
Supplement: jkae218_Supplementary_Data [file jkae218_supplementary_data.zip › Supplemental_Material_Legends_G3-2024-405349.docx]

## Supplementary Materials

**Table S1.** List of allelic QTLs obtained from 157 nonindel lines.

**Figure S1.** F1 genotyping approach.

**Figure S2.** Comparison of genomic and transcriptomic genotypes of *P. nigra* haplotypes on chromosome 1 of the F1 lines GWR_100_286.

**Figure S3.** The physical map of 507 markers used in multi-genotype QTL analysis.

**Figure S4.** Comparison of dosage QTLs observed in this article (with 343 F1 hybrids) and previous studies (with 592 F1 hybrids).

**Figure S5.** Observed QTLs for biomass-related traits with the single models.

**Figure S6.** Observed QTLs for leaf morphology traits, using the single models.

**Figure S7.** GO enrichment analysis for differentially expressed genes in allelic QTLs.

**Figure S8.** Observed QTLs for biomass traits using the combined model.

**Figure S9.** Observed QTLs for leaf morphology traits using the combined model.

**File S1.** List of phenotypic traits.

**File S2.** Information of combined model.

**File S3.** Summary of marker numbers in each step of the genotyping pipeline.

**File S4.** List of identified quantitative trait loci (QTLs).

**File S5.** List of 12 traits used for calculating phenotypic variance explained by integrated QTLs.

**File S6.** Summary of variance explained by all QTLs within a trait.

**File S7**. List of differentially expressed genes within allelic QTLs.
